# Prognostic Factors for the Development of Biochemical Recurrence after Radical Prostatectomy

**DOI:** 10.1155/2011/485189

**Published:** 2011-06-15

**Authors:** Ahmed F. Kotb, Ahmed A. Elabbady

**Affiliations:** Urology Department, Faculty of Medicine, Alexandria University, Alexandria, Egypt

## Abstract

Prostate cancer is one of the most common cancers in Western countries and is associated with a considerable risk of mortality. Biochemical recurrence following radical prostatectomy is a relatively common finding, affecting approximately 25% of cases. The aim of our paper was to identify factors that can predict the occurrence of biochemical recurrence, so the patient can be properly counselled pre- and postoperatively. Medline review of the literatures was done followed by a group discussion on the chosen publications and their valuable influence. Preoperative serum total PSA and clinical stage, together with prostatectomy Gleason grade, tumour volume, and perineural and vascular invasions, were the most important variables found to influence outcome.

## 1. Introduction

In Canada, it is estimated that 1 in 7 men will develop prostate cancer (Pca) during their lifetime, and 1 of 27 will die of it (a ratio of 1 death per 4 diagnosed cases) [1]. In 2009, approximately 192.000 men from the USA were diagnosed with prostate cancer, with an estimate of 27.000 of those men dying from it [2]. 

The Food and Drug Administration (FDA) approved PSA testing in 1986 to monitor men with prostate cancer. Since then, PSA was widely used both to screen prostate cancer and to monitor treatment response and recurrence. This resulted in early diagnosis of cases with organ localized disease amenable to definitive treatment and in early diagnosis of disease recurrence following surgery with early treatment before widespread dissemination.

The ideal therapy for clinically localized prostate cancer is still controversial. Although radical prostatectomy (RP) is a valid option, the rate of biochemical recurrence following open or laparoscopic prostatectomy is estimated to be 15–40% [3, 4].

As PSA may require up to 8 weeks to be cleared from the circulation [5], biochemical recurrence cannot be diagnosed before that time frame, following radical prostatectomy. The American Urological Association and EAU defined biochemical recurrence (BCR) following RP as an initial serum PSA of greater than or equal to 0.2 ng/mL, with a second confirmatory level of PSA greater than 0.2 ng/mL [6, 7]. Freedland et al. attempted to define the lowest PSA cut point associated with PSA progression within 1 year, along with 100% 3-year risk of progression [8]. They concluded that a 0.2 ng/mL represented the most conservative cut point to define BCR, after RP. Stephenson et al. subsequently examined the correlation between 10 definitions of biochemical recurrence with metastatic progression in a clinical cohort [9]. They proposed that biochemical recurrence should be defined as a PSA >0.4 ng/mL with a confirmatory rise. Hattab et al. defined biochemical recurrence as two consecutive serum PSA measurements >0.1 ng/mL [10]. Regardless of which definition may be better in defining BCR, the aim is to identify the perioperative factors associated with a high risk of BCR, so that patients proved to be at a higher risk of BCR can be followed more closely postoperatively and may be advised for early adjuvant treatment, after RP. Cronin et al. studied 5473 patients, who underwent RP from 1985 to 2007 [11]. An analysis of perioperative clinical and pathological parameters was done with 12 definitions of BCR. They observed that the relative risks and hazard ratios were fairly consistent between the 12 definitions examined, and the statistical inference on established prognostic factors was not impacted by the definition of BCR. So research groups using different definitions for BCR seem to reach similar conclusions regarding prognostic factors.

We discuss some factors we believe to be of important influence on the progression of prostate cancer, following its definitive management.

## 2. Preoperative Factors

### 2.1. Clinical Stage

The 2002 American Joint Committee on cancer TNM clinical staging system for prostate cancer substratified localized disease as T1 and T2. According to this committee, T1 represents disease with no abnormality on DRE and T2 represents a DRE palpable disease which is further subclassified into T2a, b, and c according to the extent of prostatic lobes affected [12]. D'Amico et al. developed a risk stratification system to predict BCR, after RP [13]. Both T1c and T2a were considered low-risk groups, whereas T2b and T2c were classified as intermediate- and high-risk groups, respectively. More recently, Billis et al. described different clinicopathological characteristics between patients with clinical stage T1c versus stages T2a and T2b [14]. Freedland et al. also showed no difference in BCR rate between patients with clinical stage T2a and T2b, with excellent 10-year progression-free survival in both groups [15]. 

Cooperberg et al. reported the San Francisco experience in prostate cancer risk assessment [16]. They demonstrated that clinical stage was not a predictive factor for biochemical recurrence in their model. Armatys et al. confirmed that cases with cT2 had worse pathological outcomes than cT1c [17]. However, no statistical difference between both groups could be found regarding BCR rate. Reese et al. reported that clinical stage in 4899 men who underwent RP for localized Pca was predictive for BCR on univariate, rather than multivariate, studies [18].

Stephenson et al. included clinical stage in their nomogram to predict BCR following RP [19]. Hashimoto et al. showed that clinical stage ≥T2a is highly significant in predicting positive surgical margin (SM) following RP [20]. In their cohort, positive SM was significantly predicting a higher rate of BCR, although they did not directly examine the association between the clinical stage and the BCR. In a study on Japanese populations, Egawa et al. found that clinical stage contributed significantly in the prediction of biochemical recurrence, in their cohort of patients [21].

We confirm DRE to be an integral part in the routine urological examination, for men above 55 years of age. Suspicious findings on DRE can predict worse outcomes following definitive management, compared to cases where diagnosis was based on high serum PSA with normal DRE findings.

### 2.2. Preoperative Serum PSA and PSA Density (PSAd)

PSA and PSAd are factors predicting BCR in most of the available nomograms and studies [16, 18, 22, 23]. Radwan et al. reported that PSAd, whether measured using U/S or the true prostate weight, was highly predicting for BCR, more than PSA [23]. Freedland et al. [24] initially reported that PSAd was a strong predictor for pathological adverse effects and BCR following RP and should be integrated in the risk stratification for Pca. In a more recent study on a larger cohort of patients, Freedland et al. found that preoperative PSAd relative to PSA provides little improvement for predicting BCR following RP [25]. Brassell et al. correlated PSA and PSAd measured using both ultrasound and the true prostate volume [26]. They reported that PSA was significantly better than PSAd in predicting BCR. 

From our experience, we believe that PSAd has no added role over PSA alone as a prognostic factor for disease progression in intermediate- and high-risk groups (PSA >10 ng/mL). On the other hand, in low-risk groups, with serum PSA <10 ng/mL, PSAd is pivotal in predicting patient outcome.

## 3. Pathological Factors

### 3.1. Gleason Grade

Gleason grade is the most widely accepted grading system for prostatic adenocarcinoma. Given the inherent sampling error of diagnostic needle biopsy and the multifocal nature of this tumor, discrepancy between Gleason score (GS) of needle biopsy and RP specimen was a common finding in the literature. King summarized 11 published series that covered over 2600 patients, in which an accurate match of grading was seen in an average of only 42% of the cases [27]. We thus believe that Gleason grade obtained from needle biopsy has a limited role in the decision making for patient management, compared to the RP Gleason grading.

Gleason score 7 should not be considered in our opinion as a single disease entity, and whenever the urologist is encountered with GS 7, decision on patient management should be done based on its primary Gleason pattern. Lau et al. studied 263 men with grade 7 RP specimens [28]. They noted that patients with primary Gleason grade 4 had a significantly higher rate of progression than patients with primary Gleason grade 3 (46% versus 33%). Sakr et al. performed the same study on 534 men, followed for a mean of 34.6 months [29]. They showed that primary grade 4 was associated with a higher rate of BCR, more than primary grade 3 (23% versus 11%, at 2-year followup).

Some studies considered the percentage of high Gleason pattern as a factor predicting a higher rate of BCR [30, 31]. On the other hand, Chan et al. reported that high Gleason pattern is not likely to be reproducible [32]. It was often difficult and time consuming and results in a prognostic effect only at its extremes (greater than 70% or less than 20% with pattern 4/5).

In our institution, we do not consider the percentage of high Gleason pattern. Nevertheless, we follow the current principals of considering cases with GS ≤6 as low risk, compared to cases with GS >6 as high risk. 

### 3.2. Tumour Volume (TV)

Data about the effect of tumour volume on BCR are contradictory in the literature. Some studies could not find a correlation between tumor volume (TV) and BCR [33, 34], whereas others considered tumour volume a high predictor factor for cancer progression [35–39]. 

Fukuhara et al. used the maximum tumour diameter (MTD) as an estimate for tumour volume, to correlate with BCR [40]. They noted that, although both PSA and MTD significantly correlated with BCR on univariate analysis, only MTD independently predicted BCR on multivariate analysis. These findings were in agreement with those of Chung et al. [41].

In our institution, we prefer measuring the maximal tumour diameter rather than the tumour volume. No data is currently available for us to judge its direct effect on the rate of BCR. We believe it to be an important variable to be included in our further studies.

### 3.3. Surgical Margin

Blute et al. reported a positive margin rate of 39% in more than 2500 patients from the Mayo series, and the BFS at 5 years was 67% and 84% in patients with positive and negative SM, respectively [42]. Similarly, Hashimoto et al. reported 5-year BFS rate of 62.6% and 81.7% for patients with positive and negative SM, respectively [43]. 

Sæther et al. failed to show SM positivity to be an independent risk factor for BCR although they could show that it had a strong trend to do so (*P* = .06) [44]. 

Lake et al. studied the disease-free survival of patients with negative surgical margin versus those with either focal (FPM) or extensive positive surgical margin (EPM) [45]. In their study, the 10-year DFS was 90%, 76%, and 53% for those with negative SM, FPM, and EPM, respectively, and that achieved statistical significance. Ochiai et al. could also show that patients with positive SM longer than 3 mm had much lower outcomes than those with shorter positive SM [46].

In our experience, a positive surgical margin is not a factor toward a significantly higher rate of BCR, although we consider it an indication for adjuvant radiotherapy.

### 3.4. Perineural Invasion (PNI)

A recent study from Italy reported that 65.7% of their prostatectomy specimens that were associated with higher Gleason grades had PNI, but not BCR [47]. Merrilees et al. detected PNI in 90% of their 105 prostatectomy specimens [48]. They also concluded that PNI was not a significant predictor for the occurrence of BCR. Miyake et al. found PNI to be of significance to predict BCR on univariate analysis but not multivariate analysis [49]. Endrizzi and Seay found PNI in their 131 patients to be a significant factor predicting BCR, with a sensitivity of 82% and a specificity of 57% [50].

Beard et al. studied the association of PNI and the relapse rate following external beam radiation [51]. They reported a significant association of PNI with higher Gleason score and a higher rate of BCR on univariate analysis. On multivariate analysis, PNI was not an independent factor predicting BCR. O'Malley et al. studied 78 patients with PNI detected by prostatic biopsy with other 78 patients with absent PNI [52]. They reported no significant effect of PNI on the rate of BCR following RP, when adjusted to GS, PSA, and pathological stage. 

### 3.5. Vascular Invasion (VI)

Van den Ouden et al. reported vascular invasion (VI) in 12% of their patients who underwent prostatectomy [53]. VI was a significant factor in predicting BCR on univariate and multivariate analysis. De la Taille et al. found VI in 12.4% of their cases and confirmed VI to be an independent significant factor predicting BCR [54]. Ferrari et al. detected VI in 18% of their patients and confirmed that VI was associated with worst pathological outcomes and was an independent factor predicting BCR on long-term followup [55]. The College of American Pathologists recommended VI to be assessed routinely in radical prostatectomy specimens [56].

### 3.6. Neuroendocrinal Differentiation

Quek et al. studied the correlation between neuroendocrinal (NE) expression, using CgA, in malignant and benign acini and recurrences [57]. They detected statistically significant association of the number of NE cells in only malignant acini and clinical recurrence. Weinstein et al. also showed that the number of malignant NE cells is of prognostic significance for patients undergoing radical prostatectomy [58]. In a multivariate analysis, May et al. [59] detected neuroendocrinal differentiation to be the second most significant predictor for biochemical progression. On the other hand, other authors did not find a significant association between NE differentiation and disease progression or biochemical failure [60, 61]. 

Although there are no solid data on the influence of neuroendocrinal differentiation on BCR and although it is currently not performed as a routine staining in our institution, we recommend staining for NE cells within the prostate as this may serve to predict biochemical recurrence as proposed by some. It may also point toward considering early chemotherapy in relapsing cases with higher degree of NE differentiation.

## 4. Conclusion

There is wide consensus that biochemical recurrence occurs in a large percentage of patients following successful radical prostatectomy. No single factor can be considered toward the prediction of recurrence. Preoperative PSA, clinical stage, prostatectomy Gleason grade, tumour volume, perineural invasion, and vascular invasion are the most important clinical and pathological parameters for assessing after radical prostatectomy. Neuroendocrinal differentiation may be an important pathological criterion to look for in a prostatectomy specimen.
